# Inhibitory Effect of *Arachis hypogaea* (Peanut) and Its Phenolics against Methylglyoxal-Derived Advanced Glycation End Product Toxicity

**DOI:** 10.3390/nu9111214

**Published:** 2017-11-04

**Authors:** Sin Hee Park, Moon Ho Do, Jae Hyuk Lee, Minsun Jeong, Oh Kyung Lim, Sun Yeou Kim

**Affiliations:** 1 Food and Drug Research Division, Gyeonggi Province Institute of Health and Environment, 95 Pajang cheon-ro, Jangan-gu, Suwon-si, Gyeonggi-do 16205, Korea; psh75@gg.go.kr; 2 College of Pharmacy, Gachon University, 191 Hambakmoe-ro, Yeonsu-gu, Incheon 21936, Korea; dvmoono@naver.com (M.H.D.); wogur6378@naver.com (J.H.L.); minsun.jeong@gachon.ac.kr (M.J.); 3 Division of Functional Food Research, Korea Food Research Institute, 245 Nongsaengmyeong-ro, Iseo-myeon, Wanju_gun, Jeollabuk-do 55365, Korea; 4 Department of Physical Medicine and Rehabilitation, Gil Medical Center, Gachon University, Inchon 21565, Korea; 5 Gachon Institute of Pharmaceutical Science, Gachon University, 191 Hambakmoe-ro, Yeonsu-gu, Incheon 21936, Korea; 6 Gachon Medical Research Institute, Gil Medical Center, Inchon 21565, Korea

**Keywords:** peanut, phenolics, advanced glycation end products (AGEs), apoptosis, mitogen-activated protein kinases (MAPKs), reactive oxygen species (ROS), diabetic complications

## Abstract

Methylglyoxal (MGO) is a highly reactive dicarbonyl compound that causes endothelial dysfunction and plays important roles in the development of diabetic complications. Peanuts are rich in energy, minerals, and antioxidants. Here, we report the potential beneficial effects of peanuts, and particularly the phenolic contents, against MGO-mediated cytotoxicity. Firstly, we optimized the extraction conditions for maximum yield of phenolics from peanuts by examining different processing methods and extraction solvents. To estimate the phenolic contents of peanut extracts, a simultaneous analysis method was developed and validated by ultra-high-performance liquid chromatography–tandem mass spectrometry. We found that roasted peanuts and their 80% methanol extracts showed the highest amount of total phenolics. Secondly, we evaluated the inhibitory effects of phenolics and peanut extracts against MGO-mediated cytotoxicity. Phenolics and peanut extracts were observed to inhibit advanced glycation end product (AGE) formation as well as to break preformed AGEs. Furthermore, pretreatment with peanut extracts significantly inhibited MGO-induced cell death and reactive oxygen species production in human umbilical vein endothelial cells. Peanut extracts prevented MGO-induced apoptosis by increasing Bcl-2 expression and decreasing Bax expression, and MGO-mediated activation of mitogen-activated protein kinases (MAPKs). In conclusion, the constituents of peanuts may prevent endothelial dysfunction and diabetic complications.

## 1. Introduction

Advanced glycation end products (AGEs) are generated via the Maillard reaction, in which non-enzymatic glycation occurs between reducing sugars and amine residues on proteins, lipids, or nucleic acids [1,2]. In vivo, the major AGEs appear to be derived from the highly reactive intermediate carbonyl compounds dicarbonyls and oxoaldehydes, including 3-deoxyglucosone, glyoxal (GO), and methylglyoxal (MGO) [3]. In particular, collagen-linked AGEs initiate oxidative reactions and interact with endothelial cells, thereby leading to vascular dysfunction [4]. Endothelial dysfunction plays a role in the development of atherosclerosis and diabetic vascular disease [5]. Thus, AGE accumulation is considered to be associated with the development of diabetic complications.

Phenolic compounds generated as secondary metabolites in plants play protective roles against UV radiation and pathogens [6]. There is also increasing evidence that plant phenolics have anti-oxidative, anti-aging, anti-diabetic, antimicrobial, and anticancer activities in cellular and animal models [7]. Dietary antioxidants with low toxicity have been suggested as a remedy for treating diabetic complications, although their therapeutic potential in humans remains to be further investigated. Based on our current scientific understanding, phenolic compounds offer considerable hope for the prevention of chronic human diseases. Plants contain appreciable quantities of bioactive phytochemicals, including phenolic compounds, which are potentially good candidates as AGE inhibitors [8,9,10].

Peanuts are rich in unsaturated fatty acids that contribute to beneficial health effects with regards to metabolic and cardiovascular disease conditions [11]. To maintain optimal metabolic control in diabetes, diets rich in high monounsaturated fatty acid (MUFA) have been suggested [12]. There is increasing evidence that the consumption of phytosterols has diverse beneficial health effects [13]. Many studies have also reported that peanuts contain several types of phenolic compounds [14]. However, whereas unsaturated fatty acids and phytosterols are known as significant contributors to the alleviation of diabetes, there has to date been limited study on the properties of the phenolic compounds found in peanuts.

In this study, we aimed to evaluate potential beneficial effects of phenolic compounds in peanuts after removing lipid. Firstly, we developed a simultaneous determination method for 12 phenolic compounds in peanuts. The phenolic contents of peanut extracts were analyzed by ultra-high-performance liquid chromatography (UHPLC) coupled with a triple quadrupole detector, and we also examined the efficacy and validity of the method. Finally, we applied the method for comparison of the phenolic contents of peanut extracted using four different solvents and processed using two different methods. It has been reported that phenolic compounds, including *p*-coumaric acid (CMA), resveratrol (RV), catechin (CT), rutin (RT), and others, could have anti-glycation activities [15]. Therefore, the second aim of this study was to investigate the protective effect of phenolic compounds extracted from peanut against AGE formation and AGE lysis, as well as AGE-induced cellular toxicity via regulation of the apoptotic pathway, including mitogen-activated protein kinases (MAPKs), Bcl-2 family member expression, and reactive oxygen species (ROS) generation. Our results showed that peanut extracts containing various phenolics that can decrease AGEs and alleviate MGO-mediated apoptosis in human umbilical vein endothelial cells (HUVECs) by regulating MAPKs, Bcl-2 family members, and ROS generation.

## 2. Materials and Methods

### 2.1. Materials

The 12 standard phenolic compounds used in this study (*trans*-cinnamic acid (CNA), (−)-epicatechin (EC), (+)-catechin (CT), caffeic acid (CA), *p*-coumaric acid (CMA), rutin (RT), isoquercitrin (IQ), trans-ferulic acid (FA), trans-resveratrol (RV), luteolin (LT), quercetin (QT), and chrysoeriol (CE)) were purchased from Extrasynthese (Genay, France). HPLC-grade n-hexane, methanol, acetonitrile, acetone, and acetic acid were obtained from Fisher Scientific (Fair Lawn, NJ, USA). Ultrapure water of 18.2 Ω was prepared using a water purification system (Barnstead International, Dubuque, IA, USA). Each of the 12 phenolic compounds was prepared by weighing an exact amount and dissolving with methanol in a 50-mL volumetric flask to prepare stock standard solutions. These standard solutions were stored at −20 °C in the dark until use. The concentration of all stock solutions was 0.5 mg/mL. A multi-compound working standard solution at a concentration of 10 mg/L was prepared by mixing together appropriate volumes of each stock solution. This was then diluted serially with methanol to prepare working standards, which were stored in screw-capped amber glass tubes at −20 °C in the dark until use.

P38, phospho-p38, extracellular signal-regulated kinase (ERK), phospho-ERK, Jun N-terminal kinase (JNK) and phospho-JNK antibodies were purchased from Cell Signaling Technology (Danvers, MA, USA). Bcl-2, Bax, P53, anti-mouse and anti-rabbit antibodies were purchased from Santa Cruz Biotech (Delaware Ave, CA, USA). HUVECs (PCS-100-010) were acquired from the American Type Culture Collection (Rockville, MD, USA). 3-(4,5-Dimethylthiazol-2-yl)-2,5-diphenyltetrazolium bromide (MTT), DCFDA fluorescent dye, MGO, GO, dimethyl sulfoxide (DMSO), and bovine serum albumin (BSA) were purchased from Sigma (St. Louis, MO, USA). Endothelial growth medium (EGM) was obtained from Lonza (Walkersville, MD, USA), and trinitrobenzenesulfonic acid (TNBS) from Thermo Scientific (Rockford, IL, USA).

### 2.2. Processing of Peanut Samples

Unprocessed shelled peanuts were purchased from four Korean provinces (Kimcheon, Yecheon, Hongcheon, and Udo Island). Peanuts were processed using two different procedures: roasting and steaming. For the roasting process, raw whole peanuts (kernels with skin) were roasted in a convection oven at 180 °C for 20 min. After roasting, the peanut kernels were cooled in a desiccator at room temperature and maintained in sealed plastic bags at −20 °C until use. For the steaming process, raw whole peanuts were steamed in steamer containing boiling water for 20 min. Raw whole peanuts were used as a control to compare the effect of the two different processing methods.

### 2.3. Extraction

Sample extraction was performed using the procedure described by Gültekin-Özgüven et al. [16] with modifications. Peanuts were finely ground using a blender (Food mixer HMF-590; Hanil Electronics, Seoul, Korea). To remove lipid in peanuts, ground peanuts (10 g) were defatted by mixing with hexane (10 g, 100 mL, 10 min 5 times) using a homogenizer (OMNI Macro ES; Omni International, Kennesaw, GA, USA). Lipid-removed peanuts were subjected to extraction using four different solvents: 100% ethanol, 70% ethanol, 80% methanol, and 80% acetone. Fifty milliliters of each solvent was added to 3 g of defatted peanut powder in a screw-capped polyethylene bottle. The phenolic compounds were extracted by ultrasound (Bransonic^®^ ultrasonic bath; Emerson, Danbury, CT, USA) for 30 min at 40 kHz. Thereafter, the extract was centrifuged (3000 rpm, 10 min, 4 °C) and the pellet was re-extracted with the same solvent. The extraction was repeated three times, and the resultant supernatants were combined. Finally, 20 mL of the combined extracts was evaporated under vacuum at 50 °C in a rotary evaporator. The dried samples were dissolved in 5 mL of methanol and filtered through a 0.2-μm PTFE syringe filter. The prepared extracts were stored in amber glass vials at −20 °C until used.

### 2.4. UPLC-MS/MS Analysis of Individual Phenolic Compounds

Analytical conditions were optimized according to Flores et al. [17] with slight modifications. In this study, we used a liquid chromatography–tandem mass spectrometry (LC-MS/MS) system to examine the phenolic compound constituents in peanuts. The application of tandem MS (MS-MS or MS) can be used to characterize individual compounds in a mixture or to identify the structure of compounds by separate ionization and fragmentation steps. Typically, an electrospray ionization (ESI) source is used as an atmospheric pressure ionizer. ESI introduces a parent ion into the mass spectrometer and then collision-induced dissociation (CID) enhances the fragmentation of the parent ion [18]. The triple quadrupole ion trap is very important because it can serve as an exceptionally high-specificity detector since a mass filter is capable of transmitting only the ion of choice.

Chromatographic analysis of polyphenols was performed using an Acquity UPLC system (Waters, Milford, MA, USA) and separation was achieved using a Cadenza CL-C18 column (100 mm × 2.0 mm, 3 µm particle size; Imtakt Co., Kyoto, Japan). Chromatographic separation was performed using a gradient elution with 0.1% acetic acid (*v/v*) in 5% acetonitrile as eluent A and 0.1% acetic acid (*v/v*) in acetonitrile as eluent B. The elution started at 100% of eluent A, which was held for 10 min, prior to being ramped to 30% of B eluent over the course of 15 min. This composition was held for 3 min before being returned to the initial condition in 2 min. The total run time was 20 min. The injection volume was 5 µL, the column temperature was maintained at 40 °C, and the flow rate was set at 0.3 mL/min.

Mass spectrometry analysis was carried out using a Waters Acquity TQD tandem quadrupole mass spectrometer (Waters, Manchester, UK). The ESI source was used in the negative mode. For MS/MS detection, the ionization source parameters were as follows: capillary voltage and the extractor voltage were set at 3.0 kV and 3.0 V, respectively; the source temperature was 150 °C and the desolvation temperature was 400 °C; the cone gas (nitrogen) and desolvation gas were set at flow rates of 50 and 750 L/h, respectively; and the collision-induced dissociation was performed using argon as the collision gas at a pressure of 3.7 × 10^−3^ mbar in the collision cell. Data acquisition was performed using Masslynx 4.1 software with Quanlynx programs (Waters, Milford, MA, USA).

### 2.5. Preparation of Glycated Bovine Serum Albumin

MGO and GO-modified BSA (MGO-BSA and GO-BSA) were produced by incubating 20 mg/mL BSA and 20 mM MGO or GO in phosphate-buffered saline (PBS; pH 7.4) in the presence of 0.02% sodium azide (pH 7.4) at 37 °C for 7 days. After incubation, the resultant AGEs were dialyzed with a 7-kDa molecular weight cut-off membrane.

### 2.6. Cell Culture

HUVECs were cultured in EGM supplemented containing 4% fetal bovine serum and 1% penicillin/streptomycin solution. Cells were cultured under standard cell culture conditions (37 °C in a humidified incubator containing 5% CO_2_). Cells were used between five and eight passages and at 90% confluency.

### 2.7. Cell Viability

The MTT assay was used for assessing cell viability. Briefly, HUVECs were seeded at 1.0 × 10^4^ cells/well in 96-well plates and incubated for 24 h at 37 °C. The cells were then pretreated with or without 1, 10, and 100 μg/mL of peanut extracts for 1 h, followed by treatment with MGO (400 µM) for 24 h. Following incubation, MTT solution was added after removing media containing compounds. The cells were incubated for 2 h at 37 °C. The medium was gently removed and the resultant formazan crystals were dissolved in 100 μL DMSO. The absorbance at 570 nm was recorded using a microplate reader (Molecular Devices, Sunnyvale, CA, USA). Cells were observed using IncuCyte ZOOM™ (Essen BioScience, Ann Arbor, MI, USA).

### 2.8. Western Blotting Analysis

To confirm alteration in the levels of proteins involved in the MAPK and apoptosis pathways, western blotting experiments were performed. Total proteins were extracted from cultured cells using PRO-PREPTM protein extraction solution (iNtRON Biotechnology, Seongnam, Korea) containing phosphatase. Concentrations of the extracted protein solutions were measured using the Bradford assay. Thereafter, equal amounts of protein were separated on 10% SDS-PAGE gels and then transferred onto nitrocellulose membranes. Membranes were incubated with blocking buffer containing 5% skim milk for 1 h at room temperature and overnight at 4 °C with primary antibodies. After washing, the membranes were incubated with conjugated secondary antibodies for 1 h at room temperature. Chemiluminescence was measured using a ChemiDoc XRS + imaging system (Bio-Rad, Hercules, CA, USA). Protein levels were quantified using Image Lab statistical software (Bio-Rad, Hercules, CA, USA).

### 2.9. Intracellular ROS Detection

Intracellular ROS were detected using DCFDA as a fluorogenic dye, which interacts with hydroxyl, peroxyl, and other ROS. The intracellular ROS scavenging activity of peanut extracts was measured using DCF-DA. Briefly, 2.0 × 10^5^ cells were dispensed into a 12-well plate and incubated overnight in a 5% CO_2_ atmosphere at 37 °C. After 24 h, cells were pre-treated with 1, 10, and 100 μg/mL of peanut extracts for 1 h, followed by incubation with MGO for 2 h, and then addition of 10 μM DCF-DA. The cells were then incubated for a further 20 min at 37 °C and washed with PBS. Cells were analyzed by flow cytometry (FACSCalibur flow cytometer; Becton Dickinson, San Jose, CA, USA).

### 2.10. Inhibitory Effects of Peanut Extracts and Constituent Phenolic Compounds on AGE Formation

The MGO-BSA and GO-BSA assay are exceptional methods for investigating inhibitors of protein glycation and were performed according to the method of Kiho et al. [19] with slight modifications. BSA (5 mg/mL) was incubated with 2 mM MGO or 2 mM GO in PBS (pH 7.4). A peanut extract or one of the 12 standard phenolic compounds (400 μM) was added shortly before incubation. Sodium azide (0.02%) was then added to the reaction mixture and the cells were incubated for 7 days. Aminoguanidine (AG; 1 mM) was used as a positive control. The formation of AGEs was determined using fluorescence at excitation/emission wavelengths of 355/460 nm with a VICTOR™ ×3 multilabel plate reader (Perkin Elmer, Waltham, MA, USA).

### 2.11. AGE Breaking Activity of Peanut Extracts and Constituent Phenolic Compounds

A TNBS assay was performed to determine AGE-breaking ability according to Habeeb et al. [20], with slight modifications. Briefly, 1 mL of MGO-BSA or GO-BSA solution (1 mg/mL) was mixed with peanut extracts (0.1, 0.5, or 1 mg/mL) or one of the 12 standard phenolic compounds (400 μM), and then incubated for 24 h. After incubation, 1 mL of 4% NaHCO3 (pH 8.5) and 1 mL of 0.1% TNBS were added and the solution was incubated at 40 °C for 2 h. After the reaction, 1 mL of 0.1% SDS and 0.5 mL of 1 N HCl were added. AGE-lysis ability was measured using a microplate reader at 340 nm (Molecular Devices, CA, USA).

### 2.12. Statistical Analyses

The data were expressed as the mean ± SD. Statistical analyses were performed using GraphPad Prism 5 (GraphPad Software, Inc., San Diego, CA, USA). Results were analyzed using one-way ANOVA followed by Bonferroni’s test. A *p*-value < 0.05 was considered statistically significant.

## 3. Results

### 3.1. Method Development for UHPLC-MS/MS Analysis

In this study, we analyzed individual phenolic compounds in the ESI (−) mode in UHPLC-MSMS, as sensitivity in the negative mode is typically superior to that in the positive mode when analyzing phenolic compounds [21]. The detailed parameters, including MS/MS transition, cone voltages, and collision energies, for each compound are shown in Table 1. Optimization of the determination conditions was carried out according to Alarcón Flores et al. [17]. Under negative ionization and full scan mode, deprotonated ions, [M–H]-, were observed for all 12 compounds. The cone voltage was gradually adjusted to obtain the most abundant parent ion; thereafter, the collision energy was optimized. Among the daughter ions fragmented by collision energies, the most sensitive transition was selected for quantification and the second one for identification. However, for CNA and CA, only one transition was observed.

The validation of the UHPLC-ESI-MS/MS method for analysis of the 12 selected phenolic compounds was accomplished by determining the following parameters: linearity, LOD (limit of detection), LOQ (limit of quantification), repeatability, and recovery (Table 2) [22,23].

Linearity was evaluated by constructing external calibration curves for each compound using a working solution containing all 12 phenolic compounds. Calibration curves were obtained by plotting the analyte peak area versus its concentration for six different concentrations. Each concentration of the mixed standard solution was evaluated in triplicate and regression parameters were calculated. The correlation coefficient of all but one analyte was above 0.99, indicating good linearity. Only chrysoeriol showed a relatively low correlation coefficient of less than 0.99. These values are shown in Table 2 together with detailed data of the regression equation.

The sensitivity of the developed method was calculated by determining the LOD and LOQ. Determination of the signal-to-noise ratio (S/N ratio) is estimated by comparing measured signals from the analysis object material with known concentrations of analyte with those of blank materials. Generally, the S/N ratio of LOD is 3 and LOQ is 10. The LOQ of an individual analytical procedure is the lowest amount of analyte in a sample that can be quantitatively determined [24]. Six replicates of the standard solutions were analyzed. LODs, based on a signal-to-noise ratio of 3:1, were achieved for the 12 phenolic compounds in the range 0.001–0.034 mg/L. LOQs, based on a signal-to-noise ratio of 10:1, between 0.003 and 0.101 mg/L were obtained.

The recoveries of phenolic compounds were estimated by spiking standard solution into the investigated matrix of peanut extracts. Recovery was calculated as follows:

(1) $$Recovery\;\left(\%\right)=\;\frac{\left(R_{1}\;-\;R_{0}\right)}{C_{spiked}}\;\times \;100$$

Before extraction, standard phenolic compounds were spiked at a level of 1 mg/kg into aliquots of peanut extract (*R*_1_). Recovery was calculated by comparing the response of an unspiked sample (*R*_0_) and spiked sample (*R*_1_). In this study, the linear ranges of CT, EC, and CMA were 0.0625~4.0 mg/L, 0.0625~2.0 mg/L, and 0.0625~2.0 mg/L, respectively. However, the initial concentrations of these three compounds calculated from standard curves were higher than their maximum linear range. Thus, the initial recovery values of these three main compounds were not acceptable. To reduce the burden of analyte, samples were diluted by 5–10 times until the diluted concentration fell within the linear range, whereupon the value was re-estimated. Using this procedure, recovery rates within the range of 83.9%~129.3% were obtained. Table 2 shows the final results.

Repeatability means precision under the same operating conditions over a given interval of time. The repeatability of the UHPLC-MS/MS method was examined by evaluating the relative standard deviations RSD (%) of the response of the peak. The RSD values of repeatability for each phenolic compound are shown in Table 2. To estimate the intra-day precision, analysis was performed six times in the same day at a known concentration (1 mg/L) of standard mixture. For inter-day precision, we also performed six replicate determinations on three non-consecutive days. For intra-day precision, the RSD values were in the range 1.48–3.09%, whereas inter-day values were in the range 0.98–10.47%. UPLC-MS/MS chromatograms of phenolics are shown in Figures S1 and S2.

### 3.2. Determination of Polyphenols in Peanut Extracts

The applicability of the proposed UHPLC-MS/MS method was evaluated for the determination of the 12 phenolic compounds in four different extracts of peanut. Peanuts collected from four different Korean provinces were extracted and the contents of phenolic compound were analyzed. The calibration curves in the validation range shown in Table 2 were used for quantification. Compounds were identified by comparison of their retention times and ion ratios of between quantification and qualification SRM transitions. According to SANTE /11945/2015 [23], in analyses using a triple quadrupole MS/MS detector, the ion ratio should correspond to that of the calibration standard when measured under the same conditions. The ion ratio should be within the range of ±30% (relative). In addition, the retention time should be within the range of ±0.1 min. All of the 12 compounds analyzed satisfied these criteria.

Generally, CT, EC, and CMA were present at the highest average concentrations in peanut extracts. The average phenolic compound contents of peanut extracts obtained using different processing procedures and different extraction solvents are shown in Table 3. CNA, CMA, RA, RV, and QT were extracted at a low level or rarely extracted in 100% ethanol. Concentrations of the target compounds that were less than the LOQ are expressed as ND (not detected).

The phenolic content (mg/kg) of cinnamic acid, epicatechin, catechin, caffeic acid, *p*-coumaric acid, rutin, isoquercitrin, trans-ferulic acid, resveratrol, luteolin, quercetin, and chrysoeriol ranged from ND to 3.9, 17.0 to 32.1, 22.0 to 41.0, ND to 1.2, 0.6 to 57.9, 2.3 to 5.4, 0.0 to 0.4, ND to 1.4, ND to 0.8, ND to 0.7, ND to 6.5, and ND to 0.9, respectively.

CMA was detected in both the raw and the processed (roasted and steamed) peanuts. Although there were differences depending on the type of extraction solvent, the levels were significantly higher (3~11 fold) in roasted peanuts and in steamed peanuts (1.5~5 fold) compared to those in raw peanuts. This may be explained by the thermolytic effect of processing, which may have caused the release of bound CMA. This was also the case for FA, CA, and CNA. RT showed the same pattern as CMA, except when extracted with 100% ethanol. In contrast, levels of EC (9–36.2%), CT (18.9–31.6%), LT (11.3–42.3%), and CE were decreased as a consequence processing (roasted and steamed). Furthermore, IQ, RV, and QT were only increased significantly in peanuts processed by roasting (*p* < 0.05).

Since the contents of the individual constituent phenolics of peanut differed depending on the processing method and the extraction solvent, the total amount of the phenolic compounds was used as a criterion for evaluating extraction efficiency. In our analysis, we found that the roasting process yields the highest phenolic content compared to raw peanuts and steamed processing. Moreover, among the different solvents, 80% methanolic extraction resulted in the highest extraction yield. Taken together, our results indicate that the total amounts of phenolics were significantly higher 80% methanolic extracts of roasted peanuts (Table 4). Because the 80% methanol extract of roasted Udo Island peanuts contained a highest content of phenolic compounds among all examined peanut extracts, we used this extract for further bioassays. Tables S1–S4 show the phenolic compound contents of peanuts collected from the four different provinces.

### 3.3. Effect of Peanut Extracts on the Formation of AGEs

Natural products and their components are generally considered to be relatively safer for human health than synthetic compounds. Moreover, nuts are known to have beneficial effects on diabetes and AGEs. We accordingly hypothesized that peanuts may have the ability to inhibit AGE formation, and tested this hypothesis by performing an AGE formation assay by measuring fluorescence.

**Figure 1:**
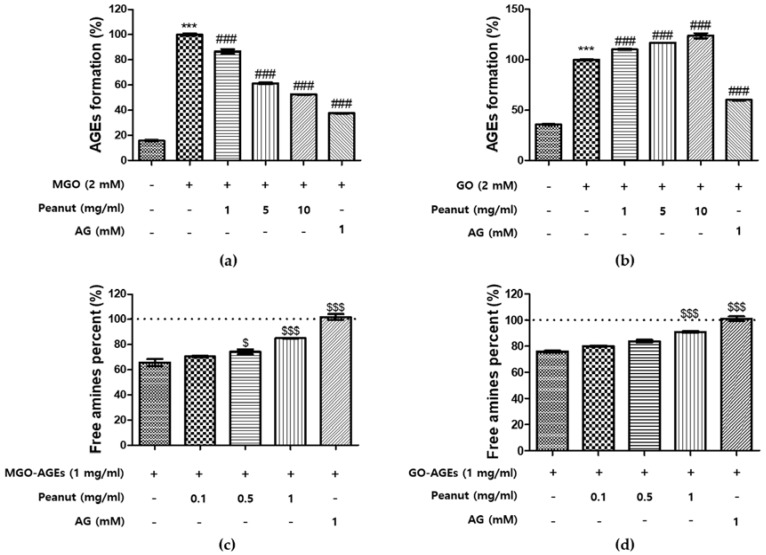
The effect of peanut extract on methylglyoxal (MGO)-induced glucotoxicity. The effects of peanut extract on in vitro advanced glycation end product (AGE) formation was examined using an AGE formation assay: MGO-mediated AGE formation (**a**); and glyoxal (GO)-mediated AGE formation (**b**). Bovine serum albumin (BSA; 5 mg/mL) was incubated with 2 mM MGO or GO in the presence or absence of each sample in phosphate-buffered saline for seven days. The AGE-breaking ability of peanut extracts was evaluated by breaking of: MGO-BSA (**c**); and GO-BSA (**d**), using the TNBS assay. The baseline frequency of BSA-free amines is represented by the dotted line at 100%. The percentage of each experiment is presented as the mean ± SD of three independent experiments. (*** *p* < 0.001 vs. control; ### *p* < 0.001 vs. MGO or GO treatment only; $ *p* < 0.05 and $$$ *p* < 0.001 vs. MGO- or GO-BSA).

BSA was incubated with MGO or GO in the presence or absence of peanut extracts and aminoguanidine (AG) was used as a positive control. In MGO-BSA formation, the addition of peanut extracts showed inhibitory effect of 7.8%, 29.6%, and 27.3% at concentrations of 1, 5, and 10 mg/mL, respectively, whereas AG had a 78.6% inhibitory effect (Figure 1a). In contrast, peanut extracts did not show an inhibitory effect with respect to GO-BSA formation, whereas AG had a 62.1% inhibitory effect (Figure 1b). Inhibitory effect was calculated using the following equation:

(2) $$Inhibitory\;effect\;\left(\%\right)=\frac{MGO\;or\;GO\;control-peanut\;extract}{\left(MGO\;or\;GO\;control-BSA\right)}\times 100$$

### 3.4. Breaking Ability of Peanut Extracts on AGEs

In diabetes and its complications, the lysis of AGEs may have a broad range of impact on the treatment. To investigate whether peanut extracts had the ability to lyse preformed AGEs, the amount of glycation remaining after exposure to extracts was quantified using the TNBS assay. Peanut extracts or AG were mixed with preformed MGO-BSA or GO-BSA solutions (1 mg/mL) and incubated for 24 h. As shown in Figure 1c, peanut extracts had MGO-BSA crosslink breaking abilities of 12.0% and 24.9% at concentrations of 0.5 and 1.0 mg/mL, respectively, whereas AG showed a 102.3% breaking effect. For GO-BSA, peanut extracts showed 17.2%, 32.8%, and 62.5% crosslink breaking abilities at concentrations of 0.1, 0.5, and 1.0 mg/mL, respectively, whereas AG had an 83.3% breaking effect (Figure 1d). The breaking effect of peanut extracts on GO-BSA was accordingly superior to that on MGO-BSA. Breaking effect was calculated using the following equation:

(3) $$Breaking\;effect\;\left(\%\right)=\frac{\left(peanut\;extract-MGO\;or\;GO\right)}{\left(BSA\;control-MGO\;or\;GO\right)}\times 100$$

### 3.5. Effect of Peanut Extracts on MGO-Induced Cytotoxicity

**Figure 2:**
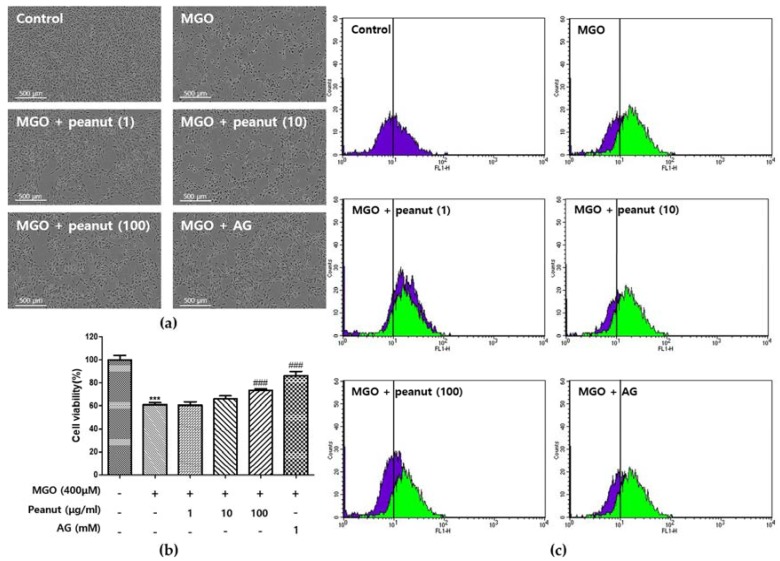
The effects of peanut extracts on methylglyoxal (MGO)-induced cytotoxicity in human umbilical vein endothelial cells (HUVECs). (**a**) Representative photographs of MGO-treated HUVECs without (−) or with (+) peanut extracts. (**b**) The viability of HUVECs treated with MGO and peanut extract. Cell viability was analyzed using the MTT assay. The percentage of cell viability is presented as the mean ± SD of three independent experiments. (**c**) The protective effect of peanut extract on MGO-induced ROS generation. HUVECs were pretreated with peanut extract for 1 h and then treated with 400 μM MGO for 2 h. ROS generation was detected by staining with the fluorescent dye DCF-DA. MGO: 400 μM methylglyoxal; MGO + peanut (1): MGO + 1 μg/mL peanut extract; MGO + peanut (10): MGO + 10 μg/mL peanut extract; MGO + peanut (100): MGO + 100 μg/mL peanut extract; MGO + AG: MGO + 1 mM aminoguanidine. (*** *p* < 0.001 vs. control; ### *p* < 0.001 vs. MGO treatment only).

Many previous studies have shown that MGO has an inhibitory effect on a number of genes related to the inflammatory response, cell cycle, apoptosis, and cell adhesion [25]. Thus, we examined the cytotoxic effect of MGO and whether peanut extracts have a protective effect. Using the MTT assay, we demonstrated that MGO treatment caused cell death (Figure 2a,b), whereas the addition of 100 μg/mL of peanut extracts significantly increased cell viability compared with the MGO treatment alone. These results suggest that peanut extracts prevent MGO-induced cytotoxicity in HUVECs.

### 3.6. Effect of Peanut Extracts on Cellular ROS Generation

It is well known that MGO can increase intracellular ROS levels and may induce cell death [26]. Therefore, we measured ROS production in HUVECs following treatment with MGO and estimated the inhibitory effect of peanut extracts. As shown in Figure 2c, MGO enhanced ROS production, whereas peanut extract pretreatment suppressed ROS production in a dose-dependent manner. This result indicated that peanut extracts can effectively inhibit MGO-induced ROS generation.

### 3.7. Effect of Peanut Extracts on MGO-Induced Apoptosis

**Figure 3:**
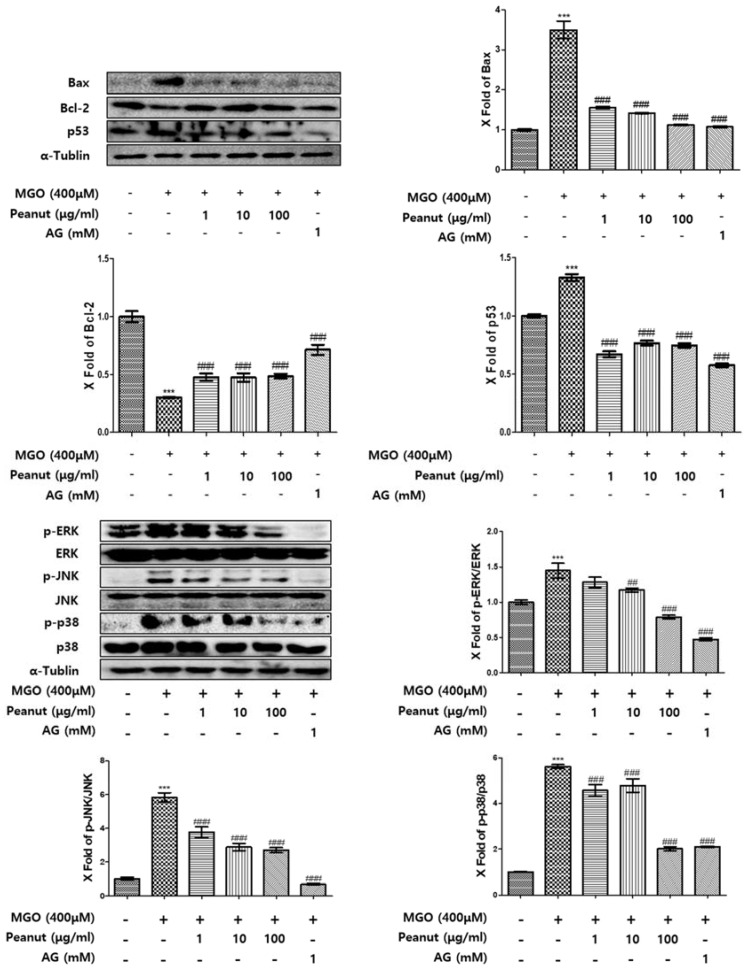
The effects of peanut extract on the expression of apoptosis-related proteins and the activation of mitogen-activated protein kinases (MAPKs). Cells were pretreated without (−) or with (+) peanut extract or aminoguanidin for 1 h, followed by treatment with 400 μM methylglyoxal for 1 h (for MAPKs) or 24 h (for Bax, Bcl-2, and p53). Bar values are presented as the mean ± SD of three independent experiments. (*** *p* < 0.001 vs. control; # *p* < 0.05, ## *p* < 0.01, and ### *p* < 0.001 vs. methylglyoxal treatment only).

Several studies have shown that MGO-induced apoptosis is related to MAPK pathway activation [27]. In western blotting, we observed that MGO activates the intracellular MAPK pathway, including ERK, JNK, and P38. MGO treatment significantly increased ERK, JNK, and P38 activation compared with the control (Figure 3). However, the phosphorylated forms of these MAPK family molecules were significantly reduced by peanut extract pretreatment in a dose-dependent manner. In addition, we investigated whether MGO and peanut extracts can affect the expression of the mitochondrial apoptotic pathway-related Bcl-2 family members, including Bcl-2, Bax, and P53, in HUVECs. As shown in Figure 3, MGO treatment increased the stress-activated protein P53 and the pro-apoptotic protein Bax, whereas it decreased the anti-apoptotic protein Bcl-2. However, peanut extract pretreatment decreased P53 and Bax proteins and increased Bcl-2. These results indicate that treatment with peanut extracts reduces apoptosis induced by MGO via suppression of the MAPK pathway and by altering Bcl-2 family expression.

### 3.8. Effect of Phenolic Compounds on the Formation of AGEs

**Figure 4:**
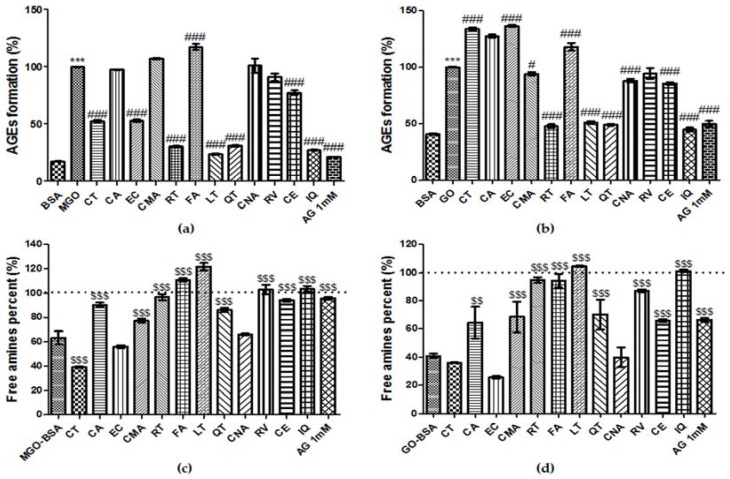
The effects of phenolic compounds on methylglyoxal (MGO)-induced glucotoxicity. The effects of phenolic compounds on in vitro advanced glycation end product (AGE) formation were examined using an AGE formation assay: MGO-mediated AGE formation (**a**); and glyoxal (GO)-mediated AGE formation (**b**). Bovine serum albumin (BSA; mg/mL) was incubated with 2 mM MGO or GO in the presence or absence of each sample (400 μM) in phosphate-buffered saline for 7 days. The AGE-breaking ability of phenolic compounds was evaluated by breaking of: MGO-BSA (**c**); and GO-BSA (**d**), using the TNBS assay. The baseline frequency of BSA free amines is represented by the dotted line at 100%. The percentage values of each experiment are presented as the mean ± SD of three independent experiments. (*** *p* < 0.001 vs. control; # *p* < 0.05 and ### *p* < 0.001 vs. MGO or GO treatment only; $$ *p* < 0.01 and $$$ *p* < 0.001 vs. MGO- or GO-BSA).

As shown in Figure 4a,b, fluorescence was significantly increased concomitant with MGO-BSA or GO-BSA formation. For MGO-BSA formation, CT, EC, RT, LT, QT, IQ, and CE at a concentration of 400 µM had inhibitory effects of 57.7%, 56.7%, 84.4%, 92.2%, 83.7%, 88.0%, and 27.0, respectively. For GO-BSA formation, 400 µM of RT, LT, QT, IQ, and CE had inhibitory effects of 88.0%, 82.4%, 85.9%, 93.0%, and 23.9%, respectively. Thus, RT, LT, QT, IQ, and CE had inhibitory effects on both MGO-BSA and GO-BSA formation. Moreover, RT, LT, QT, and IQ were almost as effective as AG in this respect.

### 3.9. Breaking Ability of Phenolic Compounds on AGEs

Phenolics and AG were incubated with pre-formed MGO-BSA or GO-BSA solution (1 mg/mL) and incubated for 24 h. For MGO-BSA breaking effect, Figure 4c shows that CA, CMA, RT, FA, LT, QT, RV, IQ, CE, and AG at a concentration of 400 µM had breaking effects of 73.6%, 38.0%, 91.0%, 129.7%, 158.9%, 62.0%, 108.0%, 109.4%, 83.7%, and 88.6%, respectively. For GO-BSA breaking effect, CA, CMA, RT, FA, LT, QT, RV, IQ, CE, and AG had breaking effects of 40.0%, 46.7%, 91.3%, 90.2%, 107.2%, 49.7%, 78.2%, 102.0%, 65.8%, and 69.0%, respectively (Figure 4d). CA, CMA, RT, FA, LT, QT, RV, IQ, and CE had breaking effects on both MGO-BSA and GO-BSA. Notably, FA, LT, RV, and IQ were more potent AGE breakers than AG.

### 3.10. Effect of Phenolic Compounds on MGO-Induced Cytotoxicity

**Figure 5:**
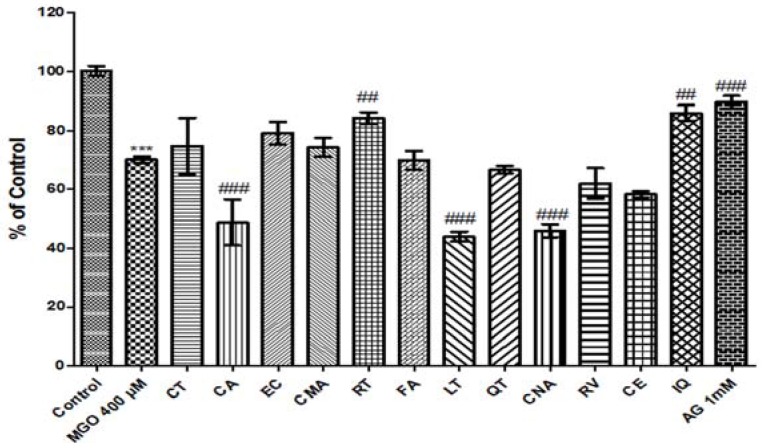
Protective effect of phenolics on methylglyoxal (MGO)-induced cell death. Human umbilical vein endothelial cells were pretreated with 100 μM of phenolic compound for 1 h, followed by MGO treatment (400 μM). Bar values are presented as the mean ± SD of three independent experiments. (*** *p* < 0.001 vs. control; ## *p* < 0.01 and ### *p* < 0.001 vs. MGO treatment only).

We demonstrated the cytoprotective effect of various phenolic compounds in peanut extracts at different concentrations. HUVECs were pretreated with 1, 10, and 100 μM phenolic compounds for 1 h, followed by treatment with MGO (400 μM). To estimate the effects of phenolics on MGO-induced cytotoxicity in HUVECs, we performed an MTT assay. None of the 1 and 10 μM samples showed a protective effect against MGO-induced cytotoxicity (data not shown). As shown in Figure 5, only RT and IQ showed a slight protective effect.

## 4. Discussion

The phytochemical properties of peanuts, including isoflavones, flavonoids, resveratrol, phenolic acids, and tocopherol, have been intensively studied in recent years [28,29,30]. However, there have been no reports of changes in the contents of the 12 phenolic compounds examined in the present study in peanuts following extraction and processing. In this study, we successfully generated peanut extracts rich in phenolic compounds using hexane, which is the most commonly used and preferred solvent for oilseed extraction [31].

We found that the gross contents of phenolic compounds in Udo Island peanuts were the most abundant among the four regional samples we examined. In particular, the resveratrol content of Udo Island peanuts (ND to 3.17 mg/kg) was demonstrably higher than that of the other regional samples (ND to 0.26 mg/kg). However, the seed size of Udo Island peanuts was observed to be only one-third that of the other peanut samples examined, indicating that Udo Island peanut seeds have a larger surface area relative to volume compared with the other samples. Because peanut skin is known to be rich in phenolic content [32], the smaller seed size seems to contribute to the greater abundance of phenolic compounds in Udo Island peanuts. The contents of phenolic compounds in the peanuts sampled from the four different provinces were found to show a wide variation. Although the reason for the observed differences is not known, the cultivar of samples used and/or the cultural environment might have an effect on phenolic contents [33,34].

Generally, the peanuts consumed by humans have undergone some type of processing. This is because, when peanuts are roasted or steamed, the characteristic unpleasant taste of peanuts is removed and the sweet taste is increased. In addition, studies have shown that thermally processed fruits and vegetables have higher biological activities because of chemical changes that occur during heat treatment [35,36].

Phenolic compounds occur in nature in free or bound forms. According to Xu and Chang, thermal treatment might result in a greater availability of plant phenolic compounds in the matrix [37]. In the present study, steaming and roasting were conducted at temperatures of 100 °C and 180 °C, respectively. The gross phenolic content was found to be higher following roasting than with steaming. These results indicate that a higher processing temperature contributed to a higher release of phenolic compounds. However, although the total amount of phenolic compounds was increased, the amounts of certain individual phenolic compounds (CT, EC, LT, and CE) were reduced after thermal processing.

Chandrasekara and Shahidi showed that high-temperature (130 °C, 33 min) treatment of cashew nuts resulted in a higher total phenol content (TPC) and antioxidant activity than low-temperature (70 °C, 6 h) treatment [38]. In addition, syringic acid, gallic acid, *p*-coumaric acid, catechin, epicatechin, and epigallocatechin were increased by processing at high temperature. Furthermore, Yu et al. showed that the roasting process (175 °C for 5 min) increased the TPC of peanut skin by 40% compared to the raw peanut skin [39]. However, in the case of black beans, catechin and epicatechin losses of approximately 49% and 17%, respectively, were detected following thermal processing (boiling, 80 min). In addition, boiling treatment has been shown to cause a greater reduction in TPC and total flavonoid content than steam processing [40]. Collectively, these previous studies indicate that the phenolic content after thermal processing might differ according to material and experimental conditions.

Polar solvents are generally used for extracting polyphenols with different chemical structures and polarities from plants. Aqueous mixtures of ethanol, methanol, and acetone are frequently used solvents for polyphenol extraction. Ethanol is known to be safe for human consumption. Methanol is considered to be more efficient for the extraction of lower molecular weight polyphenols, whereas aqueous acetone is suitable for extraction of higher molecular weight flavanols [41]. Accordingly, in the present study, we employed extraction yield as a measure of a solvent’s extraction efficiency. Among the four extraction solvents we examined (100% ethanol, 70% ethanol, 80% methanol, and 80% acetone), 80% methanol was found to be the most effective in terms of extraction yield, whereas absolute alcohol was the least effective. We confirmed that CMA, CT, and EC are the main phenolic compounds in peanut extracts, accounting for over 80% of total phenolics. Very low levels of CNA, CA, FA, and CMA were extracted with absolute ethanol. These results are consistent with the findings of similar studies conducted using macadamia skin waste. Dailey and Vuong showed that the type of extraction solvent used significantly affected the recovery yields of phenolics from macadamia skin [42]. The combination of organic solvents such as methanol, ethanol, acetonitrile, and acetone with water (50%, *v/v*) resulted in the highest recovery yields for TPC and flavonoids, followed by those obtained using absolute methanol, and then water. Absolute ethanol, acetonitrile, and acetone produced the lowest recovery yields.

AGE crosslinking on collagen and elastin leads to increased stiffness of the blood vessel system. AGEs promote an increased accumulation and continued crosslinking of collagen leading to a loss of flexibility [43]. Furthermore, in diabetes, AGEs have been demonstrated to cause diabetic vascular injury [44]. In this regard, there has been an increasing interest in the use of natural plant compounds as anti-glycation agents. Many studies have reported that diabetic complications can be ameliorated by using polyphenols via the control of AGEs [45]. Moreover, inhibition of MGO- or GO-induced AGE formation has been found to play an important role in curing diabetes [46]. In the present study, the effects of peanut extracts and their constituent polyphenols on diabetes were investigated using AGE-related glucose toxicity experiments. As shown in Figure 1, treatment with peanut extracts resulted in an increase of free amines and a decrease in fluorescence in a dose-dependent manner. Phenolics in peanut extracts also showed AGE-breaking ability (Figure 4c,d). The rank order of phenolic constituents in this regard was: LT > FA > IQ > RT > RV > AG > CE > CA > QT > CMA. Among these, LT, FA, IQ, RT, and RV were more effective than the positive control (AG). Phenolic compounds containing a catecholic moiety (such as CA) are the most powerful scavengers of free radicals and they may be used as effective chain-breaking antioxidants [47]. FA, CNA, and CMA are structurally similar to CA and therefore may also have AGE-breaking activity. Our results show that these phenolic compounds have AGE breaking ability, and that among them, CMA is relatively weaker than the others (Figure 4c,d). Although CMA had a relatively weak AGE-breaking activity, it comprised the highest proportion (36.3%) of total phenolic compounds in peanut extracts. Thus, we assumed that CMA might play an important role in the AGE-breaking activity of peanut extracts. AGE inhibitory activities have been shown to increase in strength with an increasing number of hydroxyl groups on positions 3,4′ hydroxyl group flavonoids [48]. Moreover, several studies have reported that positions 6 and 8 of the polyphenol A-ring are the major active sites for trapping MGO [45]. CT, EC, QT, IQ, CE, RT, and LT have hydroxyl groups at the 3’, 4′ in the B ring and 7 positions, and the 6 and 8 positions in the A-ring as active sites. Based on these characteristics, the anti-glycation activity of phenolic compounds could be predicted and our data showed that these compounds have inhibitory effects on AGE formation and/or an ability to break AGE crosslinks (Figure 4). Furthermore, RT, LT, and RV have a strong AGE-breaking ability, which is as high as that of AG. Among these, CT and EC constitute more than 45% of the total phenolic content in peanut extracts. Thus, CT and EC might play important roles in the anti-glycation activity of peanut extracts. Previous studies have suggested that AGE formation and breakdown can occur through various mechanisms including antioxidant effects, aldose reductase inhibition, carbonyl trapping, crosslink breaking, and inhibition of non-enzymatic glycation [49]. The efficacy of phenolic compounds in peanuts to inhibit non-enzymatic glycation and to break AGE crosslinks may be mediated by multiple events, potentially in relation to their antioxidative activities [7].

MGO is known to be a highly reactive metabolite of glucose that induces cellular injury and apoptosis in endothelial cells. To evaluate the cytoprotective effects of peanut extracts and the constituent phenolic compounds, we treated HUVECs with MGO (Figure 2b and Figure 5). However, with the exception of RT and IQ, the individual components of peanut extracts did not show cytoprotective effects in HUVECs. It can accordingly be assumed that the cytoprotective effects of peanut extracts are attributable to positive interactions such as pharmacodynamic synergy, pharmacokinetic interactions, and complementary mechanisms of action [50]. Protection of endothelial cells from cytotoxic cell death might be one of the therapeutic implications of peanut-induced anti-diabetic and anti-atherosclerotic effects.

MAPKs play a major role in cell differentiation and cell apoptosis [51], and it has been reported that MGO induces phosphorylation of ERK, JNK, and P38 in the MAPK pathway [52]. In this study, we observed that MGO treatment induced phosphorylation of MAPKs, whereas pretreatment with peanut extract decreased MGO-mediated MAPK activation in a dose-dependent manner. These data thus support the supposition that peanut extracts have protective effects against MGO-induced apoptosis in HUVECs. ROS are involved in the Maillard reaction and the formation of free radicals occurs in the early stages of AGE formation [53]. MGO can increase ROS generation, and ROS generation may play a role in AGE-RAGE interaction [25]. Figure 2c shows that MGO treatment increased ROS production in HUVECs, whereas pretreatment with peanut extracts ameliorated ROS production dose dependently. Bcl-2 family member proteins also play critical roles in regulating the process of apoptosis [54]. Bax and Bcl-2 located in the mitochondrial membrane are transcriptional targets for the tumor suppressor protein, P53, which induces cell apoptosis and regulates mitochondrial function and oxidative stress [55]. In the present study, treatment of HUVECs with 400 μM MGO decreased the expression of Bcl-2 but increased that of Bax and P53. However, pretreatment with peanut extract attenuated the MGO-induced increase in Bax and P53 expression, and increased Bcl-2 expression. In consideration of these data, we suggest that peanut extracts are likely to protect HUVECs via regulation of the MAPK pathway, Bcl-2 family members, and ROS production. In the present study, we found that the amount and type of phenolics differ according to the pretreatment of peanuts or extraction solvent. In roasted or steamed peanuts, the major phenolic compounds, catechin and epicatechin, are reduced by roasting, whereas the amount of coumaric acid, another major phenolic compound, is significantly increased. In addition, the amounts of rutin, quercetin, and isoquercitrin, which are highly effective among the minor phenolics, were also increased. We accordingly assumed that the efficacy of roasted peanuts extracted with 80% MeOH, in which phenolics change and which yield high amounts of phenolics, will be higher than that of raw or steamed peanuts. Peanuts are a rich source of fatty acids, fiber, phytosterols, and phenolic compounds, which contribute to the health benefits of these nuts [56]. It has been reported that the regular consumption of peanuts may confer a lower risk of cardiovascular disease and decrease inflammatory markers in adult humans [57,58]. Moreover, we have shown that peanut extracts have anti-glycation and cytoprotective effects in HUVECs. When all these facts are considered, we anticipate that use of peanuts as a dietary resource for polyphenols will increase in the future.

## 5. Conclusions

In this study, we confirmed that phenolic-rich peanut extracts not only inhibit AGE formation in the presence of MGO or GO, but also possess the ability to break preformed AGEs. These effects may be attributable to the antioxidative activities of phenolic compounds [7], or to the unknown mechanisms that can affect different steps in AGE formation and breakdown [49]. Furthermore, peanut extracts decreased MGO-induced cell death by reducing ROS, suppressing MAPK activation, and regulating the expression levels of Bcl-2 family members. Consequently, peanut extracts protect HUVECs from MGO-induced death. It can be suggested that increased consumption of peanuts might be an effective means of preventing glycotoxicity-induced diseases. Moreover, cytotoxicity, AGE formation, and diabetic complications may be reduced by consumption of peanuts. Nevertheless, one of the limitations of this study is that we did not examine the efficacy of the individual phenolic compounds found in peanuts, which should accordingly be evaluated in future research.

## Figures and Tables

**Table 1:** ESI-MS analysis for the 12 standard phenolic compounds.

Compound	Retention Time (min)	Cone Voltage (V)	Quantification Transition (m/z) *	Confirmation Transition (m/z) *	Ion Ratio (%)
(+)Catechin (CT)	4.35	35	289 > 245 (15)	289 > 109 (25)	75.3
Caffeic acid (CA)	4.97	25	179 > 135 (15)		
(−)Epicatechin (EC)	5.25	35	289 > 109 (20)	289 > 245 (15)	63.4
*p*-Coumaric acid (CMA)	6.31	25	163 > 119 (15)	163 > 93 (25)	5
Rutin (RT)	6.68	50	609 > 300 (35)	609 > 271 (55)	43
*trans*-Ferulic acid (FA)	6.89	25	193 > 134 (15)	193 > 149 (10)	50.5
Isoquercitrin (IQ)	6.99	45	463 > 300 (25)	463 > 271 (40)	51.4
Resveratrol (RV)	8.95	40	227 > 143 (25)	227 > 185 (18)	76.5
Luteolin (LT)	9.89	45	285 > 133 (35)	285 > 107 (35)	13
Quercetin (QT)	9.98	35	301 > 151 (22)	301 > 179 (20)	37.7
*trans*-Cinnamic acid (CNA)	10.54	30	147 > 104 (10)		
Chrysoeriol (CE)	11.53	40	299 > 284 (20)	29 > 256 (30)	23.1

* Collision energies are shown in brackets.

**Table 2:** Regression, linear range, LOD, LOQ, recoveries, and precision of the 12 phenolic compounds.

Compound	Regression	R^2^	Linear Range (mg/L)	LOQ (mg/L)	LOD (mg/L)	Recovery (%)				STD Solution Concentration (1 mg/L)						
						Ethanol	Ethanol 70%	Methanol 80%	Acetone 80%	Intra-Day (*n* = 6)		Inter-Day (Between 3 Days (*n* = 6))				
										Mean	RSD (%)	Day 1	Day 2	Day 3	Mean	RSD (%)
CNA	15189.5X + 588.1	0.9922	0.0625–2.0	0.011	0.004	109.8	103.6	107.2	102.5	1.05	2.37	1.05	1.05	1.01	1.04	2.23
EC	10442.3X - 23.9	0.9905	0.0625–2.0	0.097	0.032	99.2	97.5	104.1	99.6	1.03	2.38	1.03	1.01	1.00	1.01	1.53
CT	8964.13X + 320.6	0.9988	0.0625–4.0	0.027	0.009	100.6	107.4	106.9	109.0	1.02	3.83	1.02	1.04	1.00	1.02	1.73
CA	81988.3X + 2639.0	0.9977	0.0625–4.0	0.005	0.002	112.5	102.6	100.5	101.9	1.03	2.84	1.03	1.05	1.04	1.04	0.98
CMA	54760.3X + 2819.4	0.9924	0.0625–2.0	0.008	0.003	103.5	96.5	112.6	109.5	1.02	1.48	1.02	1.06	0.99	1.02	3.29
RT	31479.4X + 318.3	0.9933	0.0625–2.0	0.004	0.001	101.6	102.5	103.5	99.8	0.91	2.48	0.91	0.87	0.84	0.87	3.64
IQ	57551.8X + 668.4	0.9999	0.0625–4.0	0.003	0.001	108.3	114.9	102.3	95.9	1.06	3.09	1.06	1.04	0.96	1.02	5.07
FA	37480.2X + 2359.4	0.9917	0.0625–2.0	0.004	0.001	90.2	92.6	88.9	83.9	1.01	2.42	1.01	1.06	1.03	1.03	2.76
RV	21170.8X + 1208.5	0.9936	0.0625–4.0	0.005	0.002	111.0	105.2	93.5	95.6	1.04	2.62	1.04	1.02	1.01	1.03	1.63
LT	112624X + 4641.9	0.9959	0.0625–2.0	0.015	0.005	106.2	102.4	103.1	98.8	1.10	2.21	1.10	1.09	1.04	1.07	3.12
QT	53101X + 1908.0	0.9945	0.0625–2.0	0.101	0.034	102.2	95.6	95.4	102.1	1.02	2.61	1.02	1.06	1.06	1.04	2.27
CE	145257X + 9713.4	0.9887	0.0625–2.0	0.005	0.002	116.5	109.2	102.4	106.6	1.08	2.78	1.08	1.06	0.89	1.01	10.47

LOD: Limit of detection, LOQ: Limit of quantification.

**Table 3:** Average levels of 12 phenolic compounds in peanut extracts collected from four Korean provinces.

Compound	Raw				Roasted				Steamed			
	100% Ethanol	70% Ethanol	80% Methanol	80% Acetone	100% Ethanol	70% Ethanol	80% Methanol	80% Acetone	100% Ethanol	70% Ethanol	80% Methanol	80% Acetone
CNA	N.D.	0.87 ± 0.03	3.64 ± 0.02	0.13 ± 0.02	N.D.	2.39 ± 0.08 ^a^	3.87 ± 0.06 ^a^	0.77 ± 0.04 ^a^	N.D.	1.32 ± 0.05 ^a^	3.71 ± 0.02	0.28 ± 0.03
EC	22.39 ± 0.39	31.22 ± 0.44	32.07 ± 0.83	27.71 ± 0.30	20.38 ± 0.08	24.34 ± 0.69 ^b^	25.49 ± 0.75 ^b^	22.62 ± 0.10 ^b^	16.98 ± 0.57 ^b^	21.07 ± 0.42 ^b^	20.45 ± 0.78 ^b^	22.27 ± 1.57 ^b^
CT	40.83 ± 0.21	41.03 ± 0.45	40.35 ± 0.26	40.67 ± 0.19	31.96 ± 0.25	32.58 ± 0.16	32.43 ± 0.33	32.99 ± 0.28	22.04 ± 0.41	28.07 ± 0.32	30.05 ± 0.25	30.92 ± 0.50
CA	N.D.	0.46 ± 0.01	0.55 ± 0.01	0.47 ± 0.01	0.16 ± 0.01 ^a^	1.16 ± 0.01 ^a^	1.22 ± 0.02 ^a^	1.09 ± 0.01 ^a^	0.09 ± 0.01 ^a^	0.87 ± 0.01 ^a^	1.04 ± 0.01 ^a^	1.01 ± 0.00 ^a^
CMA	0.64 ± 0.02	10.43 ± 0.16	18.92 ± 0.19	8.14 ± 0.10	7.15 ± 0.07 ^a^	50.86 ± 0.38 ^a^	57.85 ± 0.61 ^a^	46.50 ± 0.62 ^a^	3.38 ± 0.04 ^a^	20.62 ± 0.62 ^a^	29.38 ± 0.59 ^a^	15.84 ± 0.29 ^a^
RT	3.85 ± 0.01	3.61 ± 0.05	3.72 ± 0.03	3.84 ± 0.03	3.41 ± 0.06 ^b^	5.36 ± 0.07 ^a^	5.38 ± 0.05 ^a^	4.99 ± 0.01 ^a^	2.34 ± 0.24 ^b^	4.15 ± 0.02 ^a^	4.41 ± 0.04 ^a^	4.24 ± 0.05 ^a^
IQ	0.30 ± 0.00	0.30 ± 0.00	0.28 ± 0.00	0.31 ± 0.00	0.37 ± 0.00 ^a^	0.33 ± 0.00 ^a^	0.36 ± 0.01 ^a^	0.33 ± 0.00 ^a^	0.02 ± 0.00 ^a^	0.21 ± 0.01 ^a^	0.26 ± 0.00	0.24 ± 0.00 ^a^
FA	N.D.	0.24 ± 0.02	0.32 ± 0.02	0.22 ± 0.01	N.D.	1.29 ± 0.03 ^a^	1.37 ± 0.01 ^a^	1.13 ± 0.02 ^a^	0.12 ± 0.01 ^a^	1.14 ± 0.02 ^a^	1.16 ± 0.03 ^a^	0.89 ± 0.01 ^a^
RV	N.D.	0.28 ± 0.01	0.33 ± 0.01	0.27 ± 0.01	N.D.	0.64 ± 0.02 ^a^	0.79 ± 0.03 ^a^	0.40 ± 0.01 ^a^	0.05 ± 0.01 ^b^	0.21 ± 0.01 ^b^	0.24 ± 0.01 ^b^	0.32 ± 0.01 ^b^
LT	N.D.	0.71 ± 0.03	0.68 ± 0.01	0.81 ± 0.02	N.D.	0.63 ± 0.02 ^b^	0.48 ± 0.01 ^b^	0.64 ± 0.02^b^	N.D.	0.41 ± 0.01 ^b^	0.43 ± 0.01 ^b^	0.61 ± 0.01 ^b^
QT	1.20 ± 0.01	2.53 ± 0.04	2.52 ± 0.05	3.49 ± 0.00	4.75 ± 0.26 ^a^	4.99 ± 0.04 ^a^	5.06 ± 0.05 ^a^	6.47 ± 0.03 ^a^	N.D.	1.92 ± 0.03 ^b^	1.90 ± 0.04 ^b^	2.63 ± 0.03 ^b^
CE	0.72 ± 0.03	0.85 ± 0.02	0.82 ± 0.02	0.91 ± 0.02	0.31 ± 0.01 ^b^	0.69 ± 0.03 ^b^	0.82 ± 0.00	0.72 ± 0.02 ^b^	N.D.	0.33 ± 0.01 ^b^	0.44 ± 0.01 ^b^	0.63 ± 0.02 ^b^

Unit: mg/kg; Values are the means ± SD of three replications. Means followed by the letter ^a^ in each column are significantly higher compared with the raw state for the same solvent (*p* < 0.05). Means followed by the letter; ^b^ in each column are significantly lower compared with the raw state for the same solvent (*p* < 0.05). ; N.D., not detected, less than LOQ.

**Table 4:** Effect of processing and extraction solvent on the total amounts of phenolic contents in peanut extracts.

		Udo Island	Yecheon	Kimcheon	Hongcheon
Raw	Ethanol	98.52 ± 1.82	62.87 ± 1.16	64.04 ± 0.66	63.96 ± 1.54
	70% Ethanol	107.43 ± 3.24	87.13 ± 1.85	80.70 ± 0.24	103.46 ± 2.25
	80% Methanol	105.14± 0.54	97.29 ± 0.83 ^b^	87.36 ± 0.40 ^b^	114.64 ± 1.28 ^b^
	80% Acetone	97.44 ± 1.51	80.54 ± 1.84	78.24 ± 0.22	91.43 ± 0.41
Roasted	Ethanol	90.29 ± 0.91	63.64 ± 1.71	68.63 ± 0.61	51.37 ± 1.19
	70% Ethanol	142.26 ± 2.71 ^a^	108.10 ± 1.61 ^a^	121.53 ± 0.55 ^a^	118.30 ± 2.09 ^a^
	80% Methanol	147.49 ± 2.19 ^a,b^	116.32 ± 1.26 ^a,b^	128.67 ± 4.38 ^a,b^	129.11 ± 0.99 ^a,b^
	80% Acetone	126.84 ± 1.69 ^a^	102.97 ± 1.01 ^a^	114.10 ± 2.12 ^a^	108.66 ± 0.65 ^a^
Steamed	Ethanol	64.94 ± 0.90	36.90 ± 1.30	50.54 ±1.87	21.96 ± 0.25
	70% Ethanol	92.49 ± 2.42	65.35 ± 1.84	78.80 ± 1.12	82.86 ± 1.56
	80% Methanol	104.21 ± 0.81 ^b^	86.96 ± 0.74	86.39 ± 1.68 ^b^	94.33 ± 1.59 ^b^
	80% Acetone	87.03 ± 0.83	82.40 ± 1.56	73.28 ± 3.64	76.36 ± 2.87

Unit: mg/kg; ^a^ highly significant (*p* > 0.001) difference with the same extraction solvent; ^b^ highly significant (*p* > 0.001) difference with the same processing.

## Supplementary Materials

The following are available online at www.mdpi.com/2072-6643/9/11/1214/s1. Figure S1: UHPLC–MS/MS chromatogram of a standard mixture of 12 phenolic compounds (2 mg/L), Figure S2. UHPLC–MS/MS chromatogram of 12 phenolic compounds found in peanut extracts, Table S1: Contents of phenolic compounds in peanuts obtained from Yecheon, Table S2: Contents of phenolic compounds in peanuts obtained from Hongcheon, Table S3: Contents of phenolic compounds in peanuts obtained from Kimcheon, Table S4: Contents of phenolic compounds in peanuts obtained from Udo Island.

## References

[1] Singh V.P., Bali A., Singh N., Jaggi A.S. (2014). Advanced glycation end products and diabetic complications. Korean J. Physiol. Pharmacol..

[2] Thorpe S.R., Baynes J.W. (1996). Role of the maillard reaction in diabetes mellitus and diseases of aging. Drugs Aging.

[3] Dyer D., Blackledge J., Katz B., Hull C., Adkisson H., Thorpe S., Lyons T., Baynes J. (1991). The maillard reaction in vivo. Z. Für Ernährungswissenschaft.

[4] Beisswenger P.J., Moore L.L., Brinck-Johnsen T., Curphey T.J. (1993). Increased collagen-linked pentosidine levels and advanced glycosylation end products in early diabetic nephropathy. J. Clin. Investig..

[5] Lee S.E., Yang H., Jeong S.I., Jin Y.-H., Park C.-S., Park Y.S. (2011). Methylglyoxal-mediated alteration of gene expression in human endothelial cells. BioChip J..

[6] Pandey K.B., Rizvi S.I. (2009). Plant polyphenols as dietary antioxidants in human health and disease. Oxid. Med. Cell. Longev..

[7] Saibabu V., Fatima Z., Khan L.A., Hameed S. (2015). Therapeutic potential of dietary phenolic acids. Adv. Pharmacol. Sci..

[8] Liu W., Ma H., Frost L., Yuan T., Dain J.A., Seeram N.P. (2014). Pomegranate phenolics inhibit formation of advanced glycation endproducts by scavenging reactive carbonyl species. Food Funct..

[9] Peng X., Zheng Z., Cheng K.-W., Shan F., Ren G.-X., Chen F., Wang M. (2008). Inhibitory effect of mung bean extract and its constituents vitexin and isovitexin on the formation of advanced glycation endproducts. Food Chem..

[10] Lunceford N., Gugliucci A. (2005). Ilex paraguariensis extracts inhibit age formation more efficiently than green tea. Fitoterapia.

[11] Kris-Etherton P.M., Hu F.B., Ros E., Sabaté J. (2008). The role of tree nuts and peanuts in the prevention of coronary heart disease: Multiple potential mechanisms. J. Nutr..

[12] Ros E. (2003). Dietary cis-monounsaturated fatty acids and metabolic control in type 2 diabetes. Am. J. Clin. Nutr..

[13] Akhtar S., Khalid N., Ahmed I., Shahzad A. (2014). Suleria HAR: Physicochemical characteristics, functional properties, and nutritional benefits of peanut oil: A review. Crit. Rev. Food Sci. Nutr..

[14] Lopes R.M., Agostini-Costa T.N.D.S., Gimenes M.A., Silveira D. (2011). Chemical composition and biological activities of arachis species. J. Agric. Food Chem..

[15] Khangholi S., Majid F.A.A., Berwary N.J.A., Ahmad F., Aziz R.B.A. (2016). The mechanisms of inhibition of advanced glycation end products formation through polyphenols in hyperglycemic condition. Planta Med..

[16] Gültekin-Özgüven M., Davarcı F., Paslı A.A., Demir N., Özçelik B. (2015). Determination of phenolic compounds by ultra high liquid chromatography-tandem mass spectrometry: Applications in nuts. LWT Food Sci. Technol..

[17] Flores M.I.A., Romero-González R., Frenich A.G., Vidal J.L.M. (2012). Analysis of phenolic compounds in olive oil by solid-phase extraction and ultra high performance liquid chromatography–tandem mass spectrometry. Food Chem..

[18] Andersen O.M., Markham K.R. (2005). Flavonoids: Chemistry, Biochemistry and Applications.

[19] Kiho T., Kato M., Usui S., Hirano K. (2005). Effect of buformin and metformin on formation of advanced glycation end products by methylglyoxal. Clin. Chim. Acta.

[20] Habeeb A.S.A. (1966). Determination of free amino groups in proteins by trinitrobenzenesulfonic acid. Anal. Biochem..

[21] Motilva M.-J., Serra A., Macià A. (2013). Analysis of food polyphenols by ultra high-performance liquid chromatography coupled to mass spectrometry: An overview. J. Chromatogr. A.

[B22-nutrients-09-01214] 22.ICH Topic Q2 (R1) Validation of Analytical Procedures: Text and Methodology. International Conference on Harmonization, **1994 (November 1996)**, 17.

[B23-nutrients-09-01214] 23.SANTE, E. Guidance document on analytical quality control and method validation procedures for pesticides residues analysis in food and feed European Commission. **2015**.

[B24-nutrients-09-01214] 24.Guideline, I.H.T. Validation of analytical procedures: Text and methodology. Q2 (R1) **2005**, 1.

[25] Figarola J.L., Singhal J., Rahbar S., Awasthi S., Singhal S.S. (2014). Lr-90 prevents methylglyoxal-induced oxidative stress and apoptosis in human endothelial cells. Apoptosis.

[26] Chan C.M., Huang D.Y., Huang Y.P., Hsu S.H., Kang L.Y., Shen C.M., Lin W.W. (2016). Methylglyoxal induces cell death through endoplasmic reticulum stress-associated ros production and mitochondrial dysfunction. J. Cell. Mol. Med..

[27] Guo Y., Zhang Y., Yang X., Lu P., Yan X., Xiao F., Zhou H., Wen C., Shi M., Lu J. (2016). Effects of methylglyoxal and glyoxalase i inhibition on breast cancer cells proliferation, invasion, and apoptosis through modulation of mapks, mmp9, and bcl-2. Cancer Boil. Ther..

[28] Chukwumah Y.C., Walker L.T., Verghese M., Bokanga M., Ogutu S., Alphonse K. (2007). Comparison of extraction methods for the quantification of selected phytochemicals in peanuts (*Arachis hypogaea*). J. Agric. Food Chem..

[29] Kim T.P.N., Thi N.V., Van P.T., Diem P.Q.N., Thuy D.N.T., That Q.T., Phi P.N.K. (2013). Phytochemical constituents and determination of resveratrol from the roots of *Arachis hypogea* L.. Am. J. Plant Sci..

[30] Kornsteiner M., Wagner K.-H., Elmadfa I. (2006). Tocopherols and total phenolics in 10 different nut types. Food Chem..

[31] Wang L., Weller C.L. (2006). Recent advances in extraction of nutraceuticals from plants. Trends Food Sci. Technol..

[32] Chukwumah Y., Walker L.T., Verghese M. (2009). Peanut skin color: A biomarker for total polyphenolic content and antioxidative capacities of peanut cultivars. Int. J. Mol. Sci..

[33] Downey M.O., Dokoozlian N.K., Krstic M.P. (2006). Cultural practice and environmental impacts on the flavonoid composition of grapes and wine: A review of recent research. Am. J. Enol. Vitic..

[34] Malheiro R., Sousa A., Casal S., Bento A., Pereira J.A. (2011). Cultivar effect on the phenolic composition and antioxidant potential of stoned table olives. Food Chem. Toxicol..

[35] Kim J.-S., Kang O.-J., Gweon O.-C. (2013). Comparison of phenolic acids and flavonoids in black garlic at different thermal processing steps. J. Funct. Foods.

[36] Dewanto V., Wu X., Adom K.K., Liu R.H. (2002). Thermal processing enhances the nutritional value of tomatoes by increasing total antioxidant activity. J. Agric. Food Chem..

[37] Xu B., Chang S.K. (2008). Total phenolics, phenolic acids, isoflavones, and anthocyanins and antioxidant properties of yellow and black soybeans as affected by thermal processing. J. Agric. Food Chem..

[38] Chandrasekara N., Shahidi F. (2011). Effect of roasting on phenolic content and antioxidant activities of whole cashew nuts, kernels, and testa. J. Agric. Food Chem..

[39] Yu J., Ahmedna M., Goktepe I. (2005). Effects of processing methods and extraction solvents on concentration and antioxidant activity of peanut skin phenolics. Food Chem..

[40] Xu B., Chang S.K. (2009). Total phenolic, phenolic acid, anthocyanin, flavan-3-ol, and flavonol profiles and antioxidant properties of pinto and black beans (*Phaseolus vulgaris* L.) as affected by thermal processing. J. Agric. Food Chem..

[41] Dai J., Mumper R.J. (2010). Plant phenolics: Extraction, analysis and their antioxidant and anticancer properties. Molecules.

[42] Dailey A., Vuong Q.V. (2015). Effect of extraction solvents on recovery of bioactive compounds and antioxidant properties from macadamia (*Macadamia tetraphylla*) skin waste. Cog. Food Agric..

[43] Janić M., Lunder M., Šabovič M. (2014). Arterial stiffness and cardiovascular therapy. BioMed Res. Int..

[44] Manigrasso M.B., Juranek J., Ramasamy R., Schmidt A.M. (2014). Unlocking the biology of rage in diabetic microvascular complications. Trends Endocrinol. Metab..

[45] Zhu D., Wang L., Zhou Q., Yan S., Li Z., Sheng J., Zhang W. (2014). (+)-catechin ameliorates diabetic nephropathy by trapping methylglyoxal in type 2 diabetic mice. Mol. Nutr. Food Res..

[46] Al Maruf A., Lip H., Wong H., O’Brien P.J. (2015). Protective effects of ferulic acid and related polyphenols against glyoxal-or methylglyoxal-induced cytotoxicity and oxidative stress in isolated rat hepatocytes. Chem.-Biol. Int..

[47] Kancheva V.D., Boranova P.V., Nechev J.T., Manolov I.I. (2010). Structure–activity relationships of new 4-hydroxy bis-coumarins as radical scavengers and chain-breaking antioxidants. Biochimie.

[48] Shimoda H., Nakamura S., Morioka M., Tanaka J., Matsuda H., Yoshikawa M. (2011). Effect of cinnamoyl and flavonol glucosides derived from cherry blossom flowers on the production of advanced glycation end products (ages) and age-induced fibroblast apoptosis. Phytother. Res..

[49] Kyselova Z., Stefek M., Bauer V. (2004). Pharmacological prevention of diabetic cataract. J. Diabetes Complicat..

[50] Rasoanaivo P., Wright C.W., Willcox M.L., Gilbert B. (2011). Whole plant extracts versus single compounds for the treatment of malaria: Synergy and positive interactions. Malar. J..

[51] Ravindran J., Gupta N., Agrawal M., Bhaskar A.B., Rao P.L. (2011). Modulation of ros/mapk signaling pathways by okadaic acid leads to cell death via, mitochondrial mediated caspase-dependent mechanism. Apoptosis.

[52] Do M.H., Kim S.N., Seo S.-Y., Yeo E.-J., Kim S.Y. (2015). Δ-tocopherol prevents methylglyoxal-induced apoptosis by reducing ros generation and inhibiting apoptotic signaling cascades in human umbilical vein endothelial cells. Food Funct..

[53] Singh D.K., Winocour P., Farrington K. (2011). Oxidative stress in early diabetic nephropathy: Fueling the fire. Nat. Rev. Endocrinol..

[54] Ashkenazi A., Fairbrother W.J., Leverson J.D., Souers A.J. (2017). From basic apoptosis discoveries to advanced selective bcl-2 family inhibitors. Nat. Rev. Drug Discov..

[55] Lin M.T., Beal M.F. (2006). Mitochondrial dysfunction and oxidative stress in neurodegenerative diseases. Nature.

[56] Emekli-Alturfan E., Kasikci E., Yarat A. (2008). Peanut (*Arachis hypogaea*) consumption improves glutathione and hdl-cholesterol levels in experimental diabetes. Phytother. Res..

[57] Jenkins D.J., Hu F.B., Tapsell L.C., Josse A.R., Kendall C.W. (2008). Possible benefit of nuts in type 2 diabetes. J. Nutr..

[58] Jiang R., Jacobs D.R., Mayer-Davis E., Szklo M., Herrington D., Jenny N.S., Kronmal R., Barr R.G. (2006). Nut and seed consumption and inflammatory markers in the multi-ethnic study of atherosclerosis. Am. J. Epidemiol..

