# Supplementary material for: Inhibitory Effect of Arachis hypogaea (Peanut) and Its Phenolics against Methylglyoxal-Derived Advanced Glycation End Product Toxicity
Source: Nutrients. 2017 Nov 4;9(11):1214. doi: 10.3390/nu9111214 (PMC5707686; doi:10.3390/nu9111214)
Supplement: Supplementary file 1 [file nutrients-09-01214-s001.pdf]

Supplementary Materials: Inhibitory Effect of Arachis hypogaea (Peanut) and Its Phenolics against Methylglyoxal-Derived Advanced Glycation End Product Toxicity

Sin Hee Park, Moon Ho Do, Jae Hyuk Lee, Minsun Jeong, Oh Kyung Lim and Sun Yeou Kim

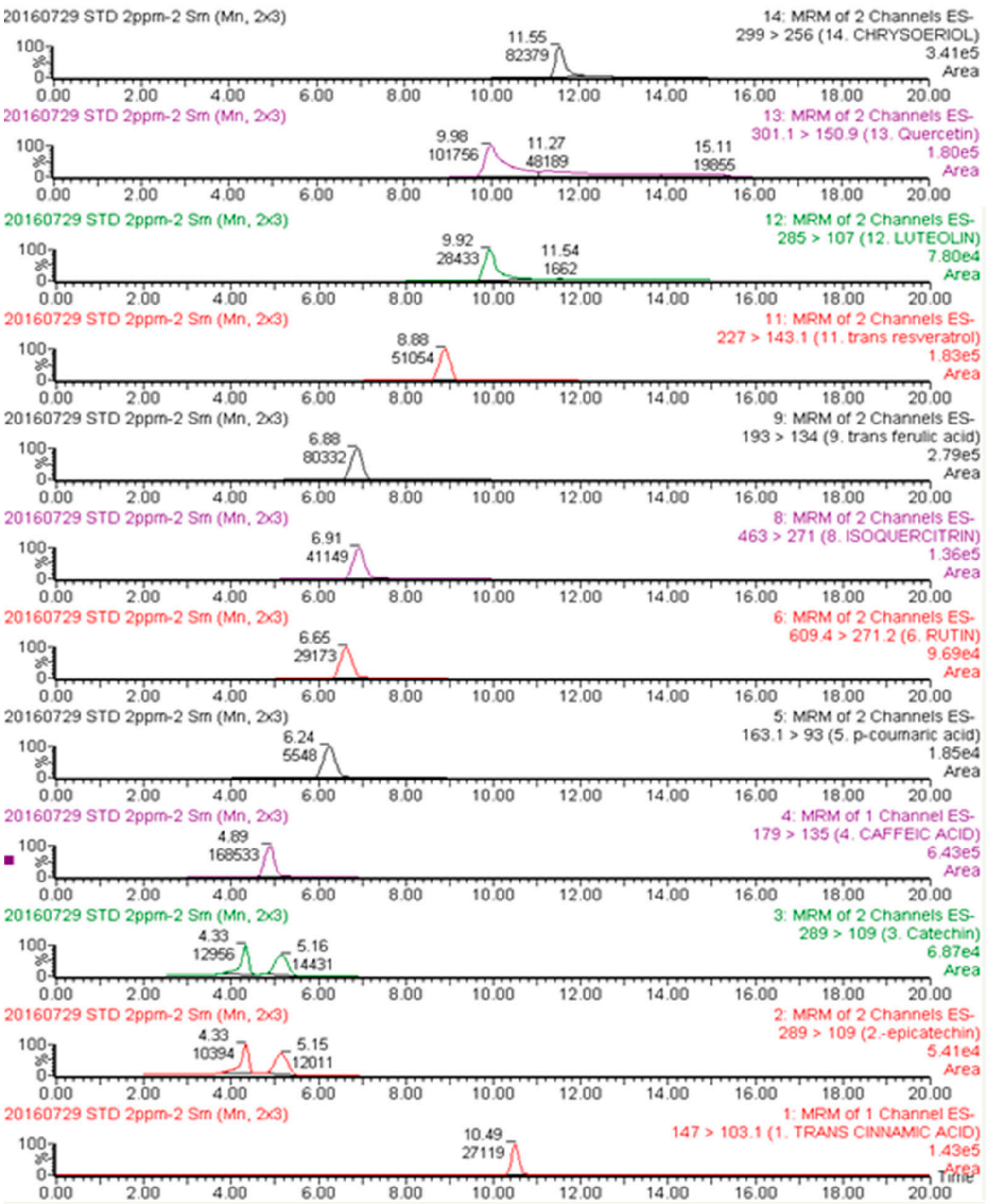

Figure S1. UHPLC–MS/MS chromatogram of standard mixture of twelve phenolic compounds (2mg/L). UHPLC-MS/MS extracted ion chromatograms of phenol standard mixture eluted with acetonitrile with 0.1% acetic acid and 5% acetonitrile with 0.1% acetic acid.

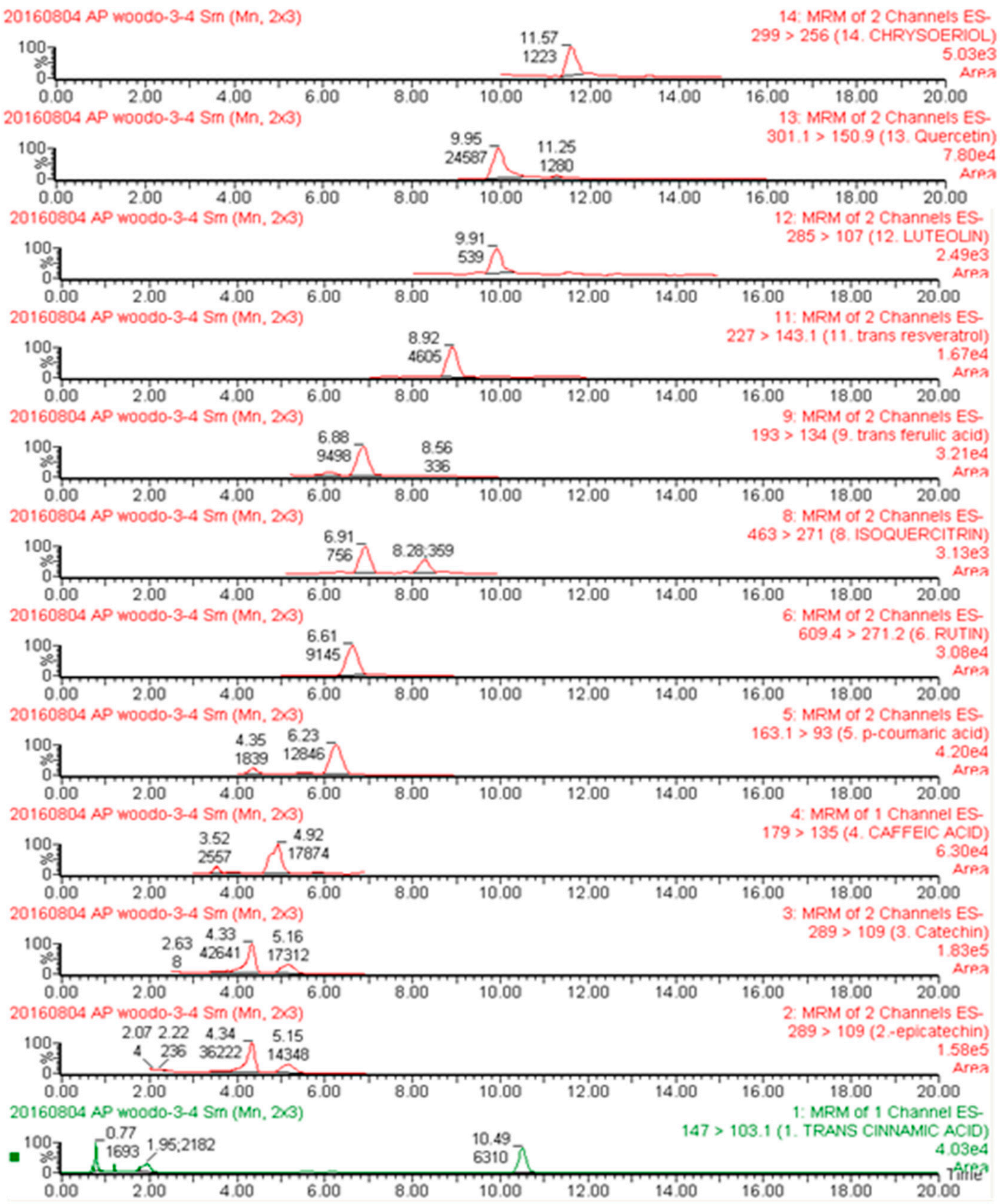

Figure S2. UHPLC–MS/MS chromatogram of peanut extract of twelve phenolic compounds.

Table S1. Contents of Phenolic compound of Yecheon.

		CNA	EC	CT	CA	CMA	RT	IQ	FA	RV	LT	QT	CE
Raw	Ethanol	N.D	24.94±1.47	33.20±1.08	N.D	0.80±0.06	2.72±0.07	0.30±0.01	N.D	N.D	N.D	0.83±0.08	0.91±0.05
	70% ethanol	1.19±0.02	29.47±0.16	33.38±1.56	0.33±0.01	16.44±0.47	2.49±0.06	0.28±0.01	0.37±0.04	0.06±0.01	0.58±0.03	1.51±0.05	1.09±0.01
	80% methanol	3.81±0.07	25.41±0.44	36.73±0.67	0.54±0.01	23.78±0.88	2.43±0.03	0.27±0.01	0.40±0.05	0.10±0.01	0.64±0.03	1.89±0.03	1.41±0.02
	80% acetone	N.D	30.02±0.67	32.99±1.10	0.29±0.03	10.32±0.33	2.56±0.04	0.18±0.00	0.32±0.03	0.02±0.01	0.63±0.02	2.02±0.07	1.22±0.01
Roasted	Ethanol	N.D	18.93±0.69	27.44±0.92	0.40±0.02	8.70±0.26	3.62±0.20	0.36±0.01	N.D	N.D	N.D	3.88±0.70	0.31±0.02
	70% ethanol	1.89±0.04	19.67±0.23	26.75±1.29	0.90±0.03	47.86±0.57	4.09±0.02	0.23±0.02	1.39±0.06	N.D	0.46±0.02	4.33±0.14	0.53±0.02
	80% methanol	3.65±0.12	18.68±0.41	25.94±0.53	1.02±0.05	56.36±1.51	4.09±0.01	0.25±0.01	1.40±0.06	N.D	0.48±0.03	4.16±0.13	0.31±0.01
	80% acetone	0.90±0.03	21.00±0.46	27.28±0.40	1.00±0.03	40.42±0.90	3.93±0.07	0.25±0.01	1.05±0.03	N.D	0.54±0.02	6.13±0.14	0.47±0.02
Steamed	Ethanol	N.D	17.34±0.24	15.35±1.41	N.D	3.62±0.20	2.49±0.02	N.D	0.11±0.01	N.D	N.D	N.D	N.D
	70% ethanol	0.68±0.02	17.01±0.17	15.43±0.17	0.24±0.01	25.29±1.71	3.44±0.11	0.06±0.01	1.36±0.07	N.D	0.26±0.02	1.61±0.06	0.24±0.02
	80% methanol	3.62±0.06	16.28±0.17	22.16±0.72	0.74±0.02	36.52±0.76	3.92±0.10	0.18±0.01	1.31±0.01	0.06±0.01	0.24±0.02	1.83±0.13	0.38±0.05
	80% acetone	0.32±0.09	27.72±0.59	26.05±0.93	0.76±0.03	20.38±1.19	3.07±0.02	0.17±0.01	1.13±0.05	0.09±0.00	0.37±0.01	1.86±0.04	0.58±0.06

Values are means ± SD for three replications.

Table S2. Contents of Phenolic compound of Hongcheon.

		CN A	EC	CT	CA	CMA	RT	IQ	FA	RV	LT	QT	CE
Raw	Ethanol	N.D	10.66±0.61	45.21±1.09	N.D	0.32±0.02	4.48±0.07	0.36±0.02	N.D	N.D	N.D	2.07±0.07	0.85±0.06
	70% ethanol	0.21±0.02	38.22±1.15	44.94±1.47	0.53±0.02	8.28±0.06	3.84±0.06	0.36±0.01	0.39±0.03	0.15±0.02	1.01±0.02	4.09±0.17	1.46±0.06
	80% methanol	3.57±0.08	36.95±0.37	44.96±1.26	0.55±0.03	16.81±0.43	3.95±0.07	0.35±0.02	0.51±0.02	0.26±0.01	1.02±0.04	4.56±0.16	1.16±0.01
	80% acetone	0.27±0.06	23.22±0.90	46.42±1.01	0.55±0.03	8.58±0.32	4.02±0.04	0.45±0.01	0.28±0.03	0.15±0.01	1.16±0.03	5.05±0.07	1.29±0.07
Roasted	Ethanol	N.D	7.47±0.45	28.60±0.75	0.18±0.03	4.81±0.20	4.54±0.08	0.27±0.01	0.05±0.01	N.D	N.D	5.08±0.12	0.43±0.03
	70% ethanol	2.70±0.17	22.34±0.37	29.68±0.95	1.11±0.04	49.63±1.58	4.22±0.17	0.27±0.02	1.65±0.11	N.D	0.66±0.07	5.27±0.22	0.78±0.08
	80% methanol	3.59±0.09	22.13±1.24	31.44±1.10	1.17±0.01	57.48±1.44	4.33±0.04	0.27±0.02	1.92±0.06	N.D	0.61±0.05	5.41±0.16	0.77±0.03
	80% acetone	1.13±0.12	14.87±0.45	31.04±0.41	1.04±0.02	45.63±0.61	4.21±0.07	0.28±0.01	1.62±0.09	N.D	0.76±0.07	7.13±0.09	0.96±0.06
Steamed	Ethanol	N.D	5.85±0.33	12.90±0.36	N.D	2.34±0.12	1.76±0.02	N.D	0.11±0.01	N.D	N.D	N.D	N.D
	70% ethanol	1.35±0.04	20.37±1.18	30.34±0.80	1.03±0.01	21.37±1.40	3.56±0.13	0.23±0.03	1.42±0.04	0.12±0.01	0.50±0.04	2.23±0.08	0.34±0.02
	80% methanol	3.74±0.06	20.88±0.68	31.20±0.52	1.19±0.01	27.95±2.21	4.15±0.11	0.27±0.01	1.71±0.08	0.18±0.02	0.49±0.02	2.10±0.03	0.48±0.03
	80% acetone	0.38±0.05	15.31±0.99	30.97±1.46	1.20±0.03	17.92±0.77	4.16±0.10	0.29±0.01	1.01±0.03	0.10±0.02	0.76±0.04	3.49±0.07	0.78±0.04

Values are means ± SD for three replications.

Table S3. Contents of Phenolic compound of Kimcheon.

		CN A	EC	CT	CA	CMA	RT	IQ	FA	RV	LT	QT	CE
Raw	Ethanol	N.D	29.99±0.61	30.59±0.58	N.D	0.51±0.04	2.62±0.05	0.34±0.01	N.D	N.D	N.D	0.85±0.06	N.D
	70% ethanol	0.95±0.09	30.41±0.73	32.20±0.56	0.24±0.01	9.50±0.23	2.66±0.05	0.35±0.02	0.12±0.01	0.21±0.02	1.02±0.08	2.45±0.09	0.59±0.02
	80% methanol	3.01±0.08	30.39±0.24	27.86±0.62	0.25±0.01	19.74±0.61	2.75±0.12	0.32±0.02	0.16±0.01	0.20±0.04	0.66±0.04	1.68±0.06	0.36±0.06
	80% acetone	N.D	31.07±0.95	31.81±1.36	0.22±0.00	6.56±0.20	2.75±0.08	0.38±0.01	0.09±0.01	0.27±0.03	1.05±0.06	3.53±0.07	0.61±0.04
Roasted	Ethanol	N.D	29.08±0.54	24.63±0.70	0.06±0.03	6.52±0.08	3.08±0.05	0.35±0.01	N.D	N.D	N.D	4.92±0.08	0.00±0.00
	70% ethanol	2.12±0.02	29.43±1.39	26.38±1.17	0.98±0.03	50.49±0.86	4.02±0.18	0.35±0.01	0.92±0.01	0.12±0.01	0.85±0.03	5.40±0.18	0.47±0.02
	80% methanol	3.28±0.25	30.90±2.03	26.16±0.61	0.81±0.04	57.15±1.72	3.92±0.12	0.34±0.00	0.85±0.06	0.00±0.00	0.36±0.04	4.25±0.03	0.63±0.02
	80% acetone	0.66±0.04	30.04±0.37	26.58±1.02	0.80±0.03	43.20±1.18	3.55±0.08	0.29±0.01	0.86±0.06	N.D	0.74±0.04	6.94±0.12	0.43±0.03
Steamed	Ethanol	N.D	22.19±1.65	24.84±0.45	N.D	3.01±0.11	2.20±0.59	N.D	N.D	N.D	N.D	N.D	N.D
	70% ethanol	1.37±0.02	25.74±0.44	25.66±0.86	0.53±0.02	20.16±0.83	3.59±0.07	0.23±0.01	0.82±0.07	0.00±0.00	0.42±0.03	1.43±0.02	0.29±0.03
	80% methanol	3.31±0.10	23.20±2.30	24.86±0.17	0.48±0.02	29.56±1.11	3.08±0.05	0.27±0.01	0.86±0.03	0.07±0.01	0.40±0.02	1.43±0.08	0.38±0.02
	80% acetone	0.22±0.02	24.84±4.18	25.45±0.35	0.49±0.03	14.35±0.66	3.80±0.12	0.23±0.02	0.81±0.03	N.D	0.58±0.04	2.13±0.12	0.38±0.01

Values are means ± SD for three replications.

Table S4. Contents of Phenolic compound of Udo island.

		CN A	EC	CT	CA	CMA	RT	IQ	FA	RV	LT	QT	CE
Raw	Ethanol	N.D	23.98±0.53	54.32±1.06	N.D	0.94±0.06	5.57±0.05	0.22±0.01	N.D	N.D	N.D	1.05±0.04	1.13±0.08
	70% ethanol	1.13±0.04	26.76±1.10	53.58±0.69	0.73±0.00	7.49±0.08	5.39±0.16	0.22±0.02	0.09±0.00	0.69±0.00	0.23±0.01	2.07±0.07	0.28±0.03
	80% methanol	4.19±0.04	35.53±2.26	51.87±1.03	0.86±0.02	15.28±0.39	5.75±0.08	0.21±0.01	0.14±0.01	0.63±0.01	0.40±0.01	1.98±0.08	0.34±0.03
	80% acetone	0.27±0.04	26.54±1.48	51.45±0.84	0.84±0.04	7.10±0.07	6.04±0.10	0.18±0.01	0.11±0.01	0.63±0.03	0.41±0.02	3.35±0.06	0.53±0.02
Roasted	Ethanol	N.D	26.02±0.57	47.17±0.25	0.01±0.01	8.59±0.25	2.39±0.08	0.50±0.02	N.D	N.D	N.D	5.12±0.13	0.50±0.02
	70% ethanol	2.84±0.19	25.91±1.87	47.51±0.91	1.64±0.05	55.45±0.84	9.12±0.19	0.49±0.02	1.22±0.06	2.45±0.05	0.56±0.04	4.97±0.13	1.01±0.06
	80% methanol	4.94±0.09	30.26±0.11	46.19±1.32	1.86±0.04	60.39±0.17	9.19±0.12	0.57±0.04	1.32±0.03	3.17±0.06	0.46±0.03	6.42±0.10	1.57±0.02
	80% acetone	0.39±0.02	24.57±0.65	47.06±0.33	1.52±0.04	56.75±2.24	8.27±0.06	0.48±0.02	0.99±0.03	1.61±0.02	0.53±0.00	5.69±0.17	1.04±0.02
Steamed	Ethanol	N.D	22.53±0.96	35.08±0.87	0.37±0.02	4.55±0.12	2.90±0.12	0.06±0.00	0.25±0.01	0.21±0.01	N.D	N.D	N.D
	70% ethanol	1.90±0.13	21.16±1.07	40.86±0.98	1.70±0.03	15.57±0.09	6.00±0.13	0.25±0.01	0.98±0.01	0.72±0.01	0.46±0.01	2.42±0.02	0.47±0.04
	80% methanol	4.16±0.07	21.42±1.02	41.96±0.82	1.76±0.04	23.49±0.82	6.50±0.16	0.32±0.01	0.77±0.01	0.65±0.02	0.59±0.03	2.26±0.13	0.52±0.01
	80% acetone	0.22±0.02	21.20±0.27	41.13±0.20	1.59±0.03	10.73±0.37	5.92±0.15	0.25±0.01	0.61±0.03	0.99±0.02	0.69±0.05	3.05±0.06	0.66±0.04

Values are means ± SD for three replications.
